# Genome of the hoverfly *Eupeodes corollae* provides insights into the evolution of predation and pollination in insects

**DOI:** 10.1186/s12915-022-01356-6

**Published:** 2022-07-06

**Authors:** He Yuan, Bojia Gao, Chao Wu, Lei Zhang, Hui Li, Yutao Xiao, Kongming Wu

**Affiliations:** 1 State Key Laboratory for Biology of Plant Diseases and Insect Pests, Institute of Plant Protection, Chinese Academy of Agricultural Sciences, Beijing, China; 2 Shenzhen Branch, Guangdong Laboratory of Lingnan Modern Agriculture, Genome Analysis Laboratory of the Ministry of Agriculture and Rural Affairs, Agricultural Genomics Institute at Shenzhen, Chinese Academy of Agricultural Sciences, Shenzhen, China; 3 Department of Entomology, College of Plant Protection, China Agricultural University, Beijing, China

**Keywords:** Hoverfly, Chromosome-level genome, Pest predation, Pollination, Digestion

## Abstract

**Background:**

Hoverflies (Diptera: Syrphidae) including *Eupeodes corollae* are important insects worldwide that provide dual ecosystem services including pest control and pollination. The larvae are dominant predators of aphids and can be used as biological control agents, and the adults are efficient pollinators. The different feeding habits of larvae and adults make hoverflies a valuable genetic resource for understanding the mechanisms underlying the evolution and adaptation to predation and pollination in insects.

**Results:**

Here, we present a 595-Mb high-quality reference genome of the hoverfly *E. corollae*, which is typical of an aphid predator and a pollinator. Comparative genomic analyses of *E. corollae* and Coccinellidae (ladybugs, aphid predators) shed light on *takeout* genes (3), which are involved in circadian rhythms and feeding behavior and might regulate the feeding behavior of *E. corollae* in a circadian manner. Genes for sugar symporter (12) and lipid transport (7) related to energy production in *E. corollae* had homologs in pollinator honeybees and were absent in predatory ladybugs. A number of classical cytochrome P450 detoxification genes, mainly CYP6 subfamily members, were greatly expanded in *E. corollae*. Notably, comparative genomic analyses of *E. corollae* and other aphidophagous hoverflies highlighted three homologous trypsins (Ecor12299, Ecor12301, Ecor2966). Transcriptome analysis showed that nine trypsins, including Ecor12299, Ecor12301, and Ecor2966, are strongly expressed at the larval stage, and 10 opsin genes, which are involved in visual perception, are significantly upregulated at the adult stage of *E. corollae*.

**Conclusions:**

The high-quality genome assembly provided new insights into the genetic basis of predation and pollination by *E. corollae* and is a valuable resource for advancing studies on genetic adaptations and evolution of hoverflies and other natural enemies.

**Supplementary Information:**

The online version contains supplementary material available at 10.1186/s12915-022-01356-6.

## Background

Aphidophagous hoverflies (Diptera: Syrphidae) are important insects for maintaining essential ecosystem services. The hoverfly *Eupeodes corollae* is a predominant aphid-specific predator and efficient pollinator in the field [1]. The larvae are important natural enemies and biological control agents for aphids, which feed on a wide range of aphid species, and have been reported to consume 3–10 trillion aphids in southern Britain each year [2, 3]. Because the larvae have limited dispersal abilities, female adults lay their eggs near plants with an aphid colony to support the maturation of the larvae, which is related to predation adaptation [4–6]. The adults feed on pollen or nectar, visit billions of flowers each year, and thus are key pollinators in natural ecosystems and agricultural crops [2, 3, 7, 8]. Several migratory hoverflies, such as *Episyrphus balteatus* and *Eupeodes corollae*, play important roles in improving pollination efficiency and maintaining hoverflies’ stable populations [9–12]. Considering that the populations of many beneficial insects, especially pollinators, are seriously declining [13, 14], hoverflies are becoming increasingly important. Moreover, larvae and adult aphidophagous hoverflies use different food sources, providing a model to study the evolution and transition of feeding habits. However, little is known about the mechanism underlying its special adaptation and evolution of predation and pollination.

Here, we present a high-quality draft assembly for *E. corollae*. Comparative genomic analysis revealed a number of gene families that likely contributed to the adaptation to predation and pollination. Moreover, numerous chemosensory genes and digestive enzymes with special or high expression levels at the larval stage were identified by transcriptomic analysis, and their function in predation and pollination is discussed. This genome assembly lays the foundation for in-depth research of *E. corollae* and will promote further analyses of predation and pollination in hoverflies and other natural enemies.

## Results

### Genome assembly and annotation of *E. corollae*

**Fig. 1:**
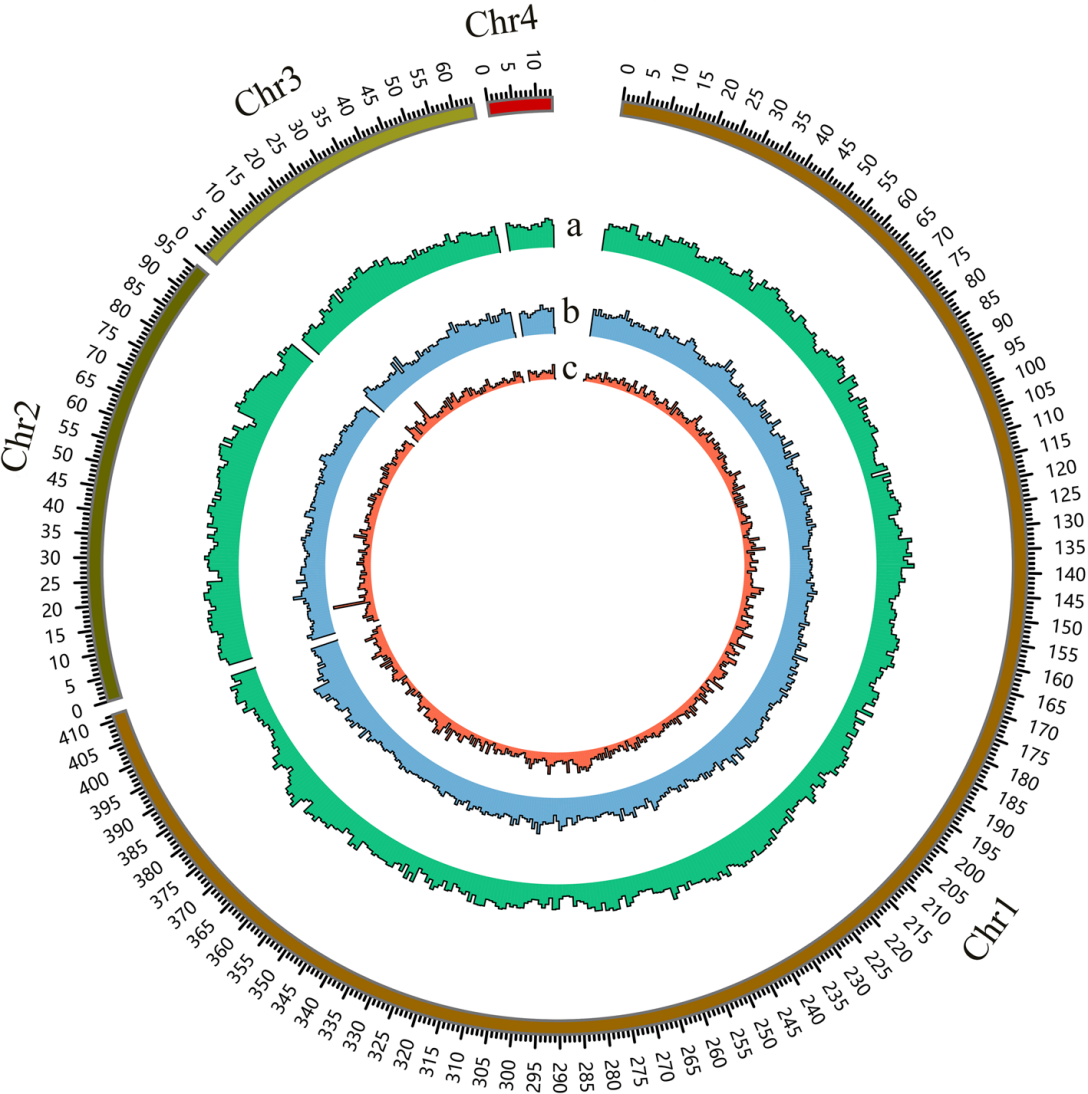
*Eupeodes corollae* genome landscape. Tracks in 1-Mb windows. **a** Distribution of GC content. **b** Repeat sequence density. **c** Gene density

In total, 60.23 Gb of clean Illumina reads were obtained after filtering (Additional file 1: Table S1). The genome size and heterozygosity of *E. corollae* were estimated by *k*-mer analysis as 604 Mb and 0.84%, respectively (Additional file 1: Fig. S1). The PacBio Sequel platform yielded 65.77 Gb (~ 109 × coverage) of high-quality data for genome assembly. De novo assembly using Wtdbg2 [15] following self-correction by CANU (version 1.8) resulted in a final genome size of 595 Mb, including 3246 contigs with an N50 length of 1.8 Mb (Table 1). According to the karyotype results (*n* = 4) published previously [16], 570.8 Mb (96.0%) of the assembled sequences were anchored into four linkage groups with a total of 55.42 Gb Hi-C clean reads (Fig. 1, Additional file 1: Table S2).

**Table 1:** Assembly statistics for the *Eupeodes corollae* genome and related statistics for three other hoverflies

Statistic	Aphidophagous		Saprophagous	
	*E. corollae*	*Scaeva pyrastri*	*Syritta pipiens*	*Eristalis tenax*
Assembled genome size (Mb)	595	320.1	318.5	487
Longest contig size (kb)	10,130	–	–	–
Number of contigs	3246	–	–	–
Contig N50 (kb)	1794	–	–	–
Number of chromosomes	4	4	5	6
GC content (%)	33.8	22.6	28.6	28
Number of gene models	23,374	32,409	19,615	27,199
BUSCO complete gene ratio (%)	97.1	99.0	98.9	98.9
Repeat (%)	51.47	30.05	30.89	47.58

We assessed the genome assembly by aligning the Illumina data with it, resulting in a mapping rate of 98.14% and a coverage rate of 97.82%. Benchmarking Universal Single-Copy Orthologs (BUSCO) analysis of the current genome identified 97.1% of the complete BUSCO genes (Additional file 1: Table S3), suggesting high integrity of the genome assembly.

We identified 306 Mb of repeat sequences, constituting 51.47% of the *E. corollae* genome (Additional file 1: Table S4). Among the repeat families, long interspersed elements (LINEs) (23.35%) were the most abundant repeat elements. In total, 23,374 gene models were predicted in the *E. corollae* genome (Table 1). For functional annotation, 16,878 (72.21%) genes had hits in the Nr database and 12,016 (51.41%) genes in the Swiss-Prot database (Additional file 1: Fig. S2).

### Gene orthology and evolution

**Fig. 2:**
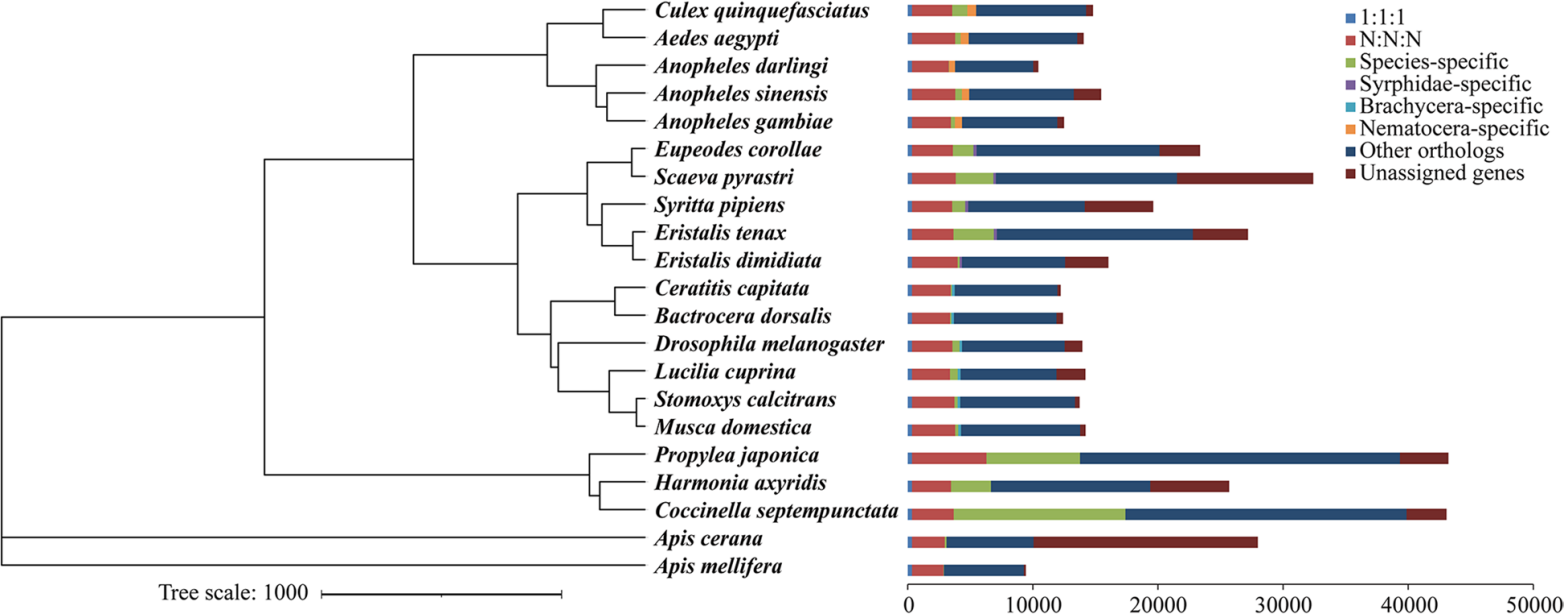
Phylogenetic relationships and gene orthology of *Eupeodes corollae* and other insects. The maximum likelihood phylogenetic tree was calculated based on 333 single-copy universal genes. The colors in the histogram indicate categories of orthology: 1:1:1, single-copy universal genes; N:N:N, multicopy universal genes; species-specific, genes without an orthologue in any other species; Syrphidae-specific, genes specific to the family of Syrphidae; Brachycera-specific, genes specific to Brachycera lineage; Nematocera-specific, genes specific to the Nematocera lineage

We compared the protein-coding genes from *E. corollae* with those of 15 dipteran insects, three coleopteran insects, and two hymenopteran insects to identify orthologous groups. Among them, 20,128 genes in the *E. corollae* genome clustered into 11,218 orthogroups (Fig. 2). The *E. corollae* genome contains 254 Syrphidae-specific genes, which were enriched in GO terms nitrogen compound metabolic process, cellular metabolic process, and cellular biosynthetic process (Fisher’s exact test, *p* < 0.05) (Additional file 2: Table S5). A total of 1640 species-specific genes were identified in the *E. corollae* genome. Gene Ontology (GO) enrichment analysis revealed that these genes were enriched in GO terms organonitrogen compound biosynthetic process and lipoprotein biosynthetic process (Fisher’s exact test, *p* < 0.05) (Additional file 2: Table S6). For the phylogenetic tree construction, 333 single-copy genes from the 20 species were used. In this analysis, *E. corollae* clustered with four other species of Syrphidae (Fig. 2). Estimations of divergence times suggest that *E. corollae* and *Scaeva pyrastri* may have diverged from their common ancestor approximately 54 million years ago (Mya).

### Comparative genomic analyses

**Fig. 3:**
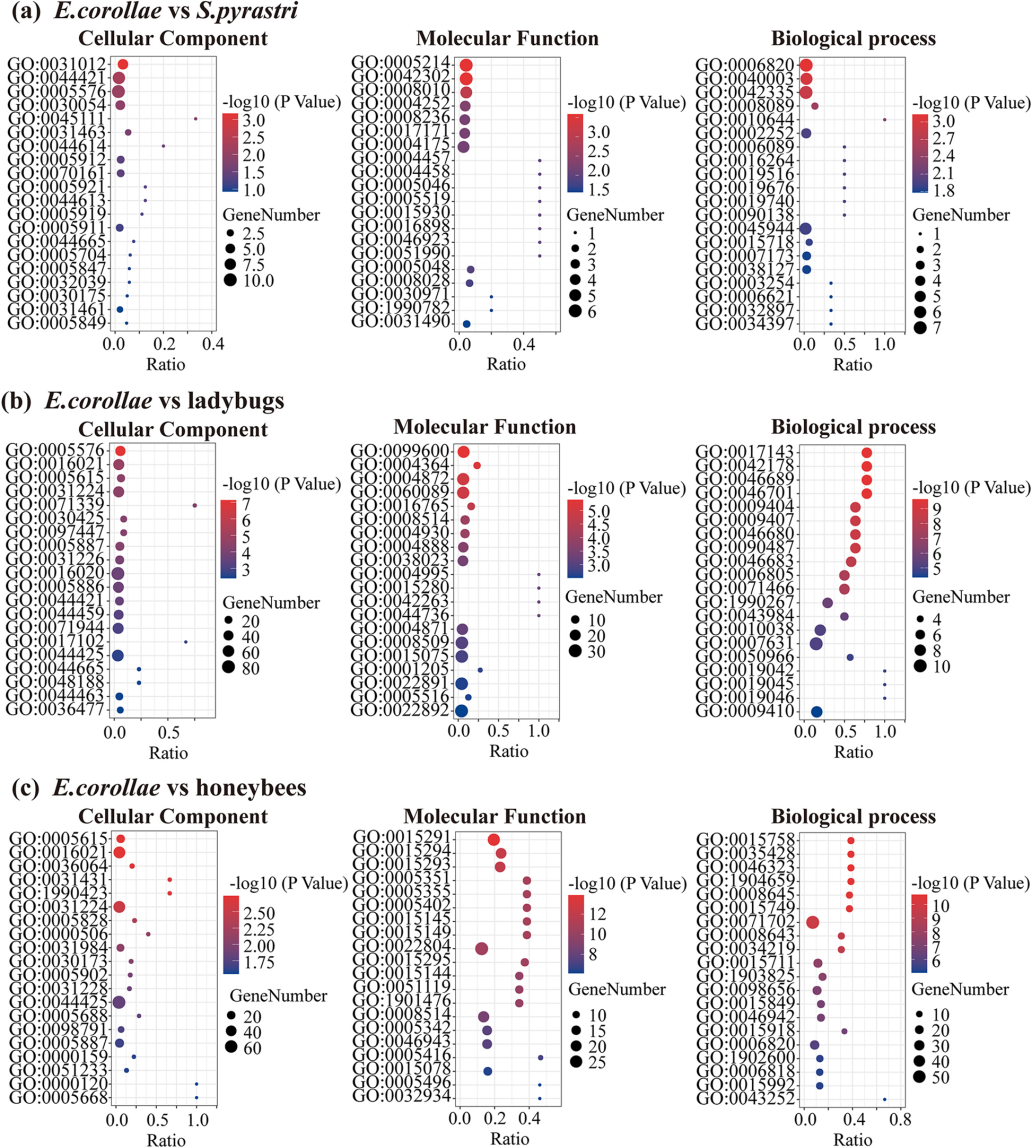
GO enrichment in proteins encoded by homology genes in *Eupeodes corollae* compared with the genome of aphidophagous hoverfly (**a**), predator ladybugs (**b**), and pollinator honeybees (**c**). GO enrichment in cellular component, molecular function, and biological process. *x*-axis: RichFactor, number of homology genes/all genes for GO term; *y*-axis: pathway name. Ladybugs: *Coccinella septempunctata*, *Harmonia axyridis*, and *Propylea japonica*; honeybees: *Apis cerana* and *Apis mellifera*

*E. corollae* and *S. pyrastri* are both aphidophagous hoverflies with similar biological characteristics and belong to the tribe Syrphini in the family Syrphidae. We compared the genome of *E. corollae* with *S. pyrastri* to uncover the mechanisms underlying its predation and pollination abilities. The 1718 homologous genes in *E. corollae* were enriched in GO terms serine hydrolase activity (GO:0,017,171) and cuticle development (GO:0,042,335) (Fisher’s exact test, *p* < 0.05) (Fig. 3a, Additional file 2: Table S7), including trypsin (4) and cuticular protein genes (5). These genes are involved in protein digestion, cuticle development, and innate immunity in insects [17, 18].

*E. corollae* and ladybugs (Coccinellidae) are both important natural predators of aphids. However, *E. corollae* larvae are monophagous insects that mainly feed on aphids, while the larvae and adults of ladybugs are polyphagous, preying on many pests such as lepidopteran larvae and aphids. In a comparative genomic analysis among *E. corollae* and three predatory ladybugs *Coccinella septempunctata*, *Harmonia axyridis*, and *Propylea japonica*, 1283 homologous genes in *E. corollae* were enriched in GO terms G-protein-coupled receptor activity (GO:0,004,930) and feeding behavior (GO:0,007,631) (Fisher’s exact test, *p* < 0.05) (Fig. 3b, Additional file 1: Fig. S3, Additional file 2: Table S8), including three gustatory receptors (GRs), which mainly involved in the perception of chemical signals, such as sugars or bitter compounds [19, 20], and three takeout-like proteins, which have been reported to play important roles in the circadian regulation and feeding response in *Drosophila* [21].

In addition, to elucidate the mechanism underlying pollination, we compared the genome of two honeybees *Apis cerana* and *Apis mellifera* with that of *E. corollae*, all of which are efficient pollinators. The 431 homologous genes in *E. corollae* were enriched in GO terms sugar:proton symporter activity (GO:0,005,351) and lipid transport (GO:0,006,869) (Fisher’s exact test, *p* < 0.01) (Fig. 3c, Additional file 1: Fig. S3, Additional file 2: Table S9), including trehalose transporter and phospholipid-transporting ATPase. These genes were found to be associated with pollination behavior and energy production during migration and might contribute to the pollination adaption in *E. corollae*.

### The genomic basis of aphid digestion

Our manual annotation of the digestive enzyme genes in the *E. corollae* genome yielded 153 serine proteases (SPs) (58 trypsin and 26 chymotrypsin), 44 carboxypeptidases, 8 α-amylases, 30 aminopeptidases, 41 phospholipases, and 36 lipases (Table 2). The large number of SPs among the digestive enzymes in *E. corollae* is consistent with the expectation that carnivorous insects have relatively greater protease activity than other insects [22]. When compared with other dipteran and coleopteran species, *E. corollae* had the fewest protease genes, which may be due to its digestion of a single-food diet such as aphids, in contrast to a broad diet of polyphagous insect species. For example, SPs were significantly expanded in the omnivorous pest *Apolygus lucorum* [23]. However, *E. corollae* had more protease genes than in honeybees, which is consistent with the honeybees’ simple diet of sugar-rich nectar (Table 2). Several digestive enzymes were arranged in tandem on the genome, including a cluster of four trypsin genes with 86.1% amino acid similarity (Ecor10293-Ecor10296), four α-amylases with 79.9% similarity (Ecor16162-Ecor16165), and 10 phospholipases with 58.9% similarity (Ecor17802-Ecor17811), suggesting that a recent replication event enhanced digestion and absorption of aphids in *E. corollae* during evolution.

**Table 2:** Number of chemosensory-related, detoxification-related, and digestion-related genes in the genome of various insects

Gene	*E. c*	*E. t*	*E. d*	*S. pi*	*S. py*	*C. s*	*H. a*	*P. j*	*A. m*	*A. c*
**Chemosensory**										
OR	46	70	35	72	48	20	28	55	141	143
IR	36	40	15	40	55	65	48	84	27	26
GR	36	66	23	56	52	25	19	50	15	13
OBP	46	71	22	41	45	32	29	78	20	17
CSP	4	3	1	3	4	28	19	47	6	5
SNMP	4	19	16	19	18	17	20	29	9	10
**Detoxification P450**										
CYP2	7	6	8	6	8	6	10	17	8	6
CYP3	31	36	24	35	30	36	42	64	28	26
CYP4	23	39	29	30	21	18	28	111	4	5
Mito	13	17	8	14	14	5	9	10	6	6
Total	74	98	69	85	73	65	89	202	46	43
**Detoxification GST**										
Delta	7	3	5	4	7	3	2	3	2	3
Epsilon	11	10	3	11	10	8	11	8	0	2
Omega	1	1	1	1	2	1	5	0	2	1
Sigma	1	1	1	1	1	1	0	2	4	5
Theta	3	4	0	3	3	2	2	3	1	0
Zeta	0	1	1	1	1	2	2	4	1	1
Microsomal	4	2	1	1	1	1	1	1	1	1
Total	27	22	12	22	25	18	23	21	11	13
**Digestion**										
Trypsin	58	103	58	103	96	66	54	127	33	32
Chymotrypsin	26	22	18	18	16	2	3	16	7	5
Carboxypeptidase	44	31	26	27	30	19	30	36	26	16
Lipase	74	58	29	52	58	69	52	96	22	26
α-Amylase	8	6	1	7	7	1	6	3	1	1
Phospholipase	41	17	11	19	28	17	18	27	26	25
Aminopeptidase	30	25	36	25	31	32	35	60	16	18

*OBP* Odorant-binding proteins, *CSP* Chemosensory proteins, *OR* Odorant receptor, *IR* Ionotropic receptor, *GR* Gustatory receptor, *SNMP* Sensory neuron membrane protein, *GST* Glutathione *S*-transferase. Abbreviations of insect species: *E. c Eupeodes corolla*, *E. t Eristalis tenax*, *E. d Eristalis dimidiate*, *S. pi Syritta pipiens*, *S. py Scaeva pyrastri*, *C. s Coccinella septempunctata*, *H. a Harmonia axyridis*, *P. j Propylea japonica*, *A. m Apis mellifera*, *A. c Apis cerana*

**Fig. 4:**
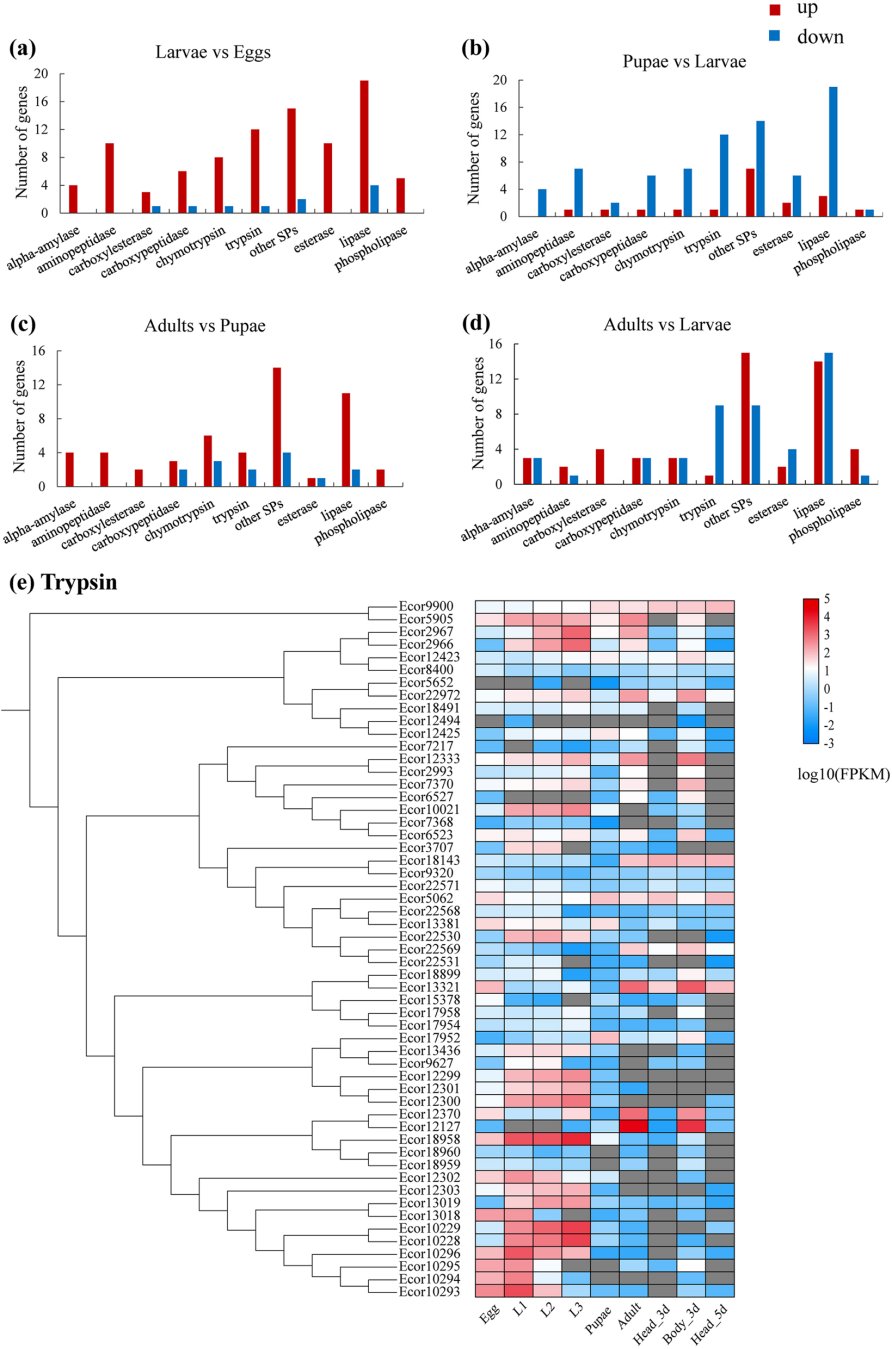
Expression profile of digestion-related genes (**a**–**d**) and phylogenetic tree for trypsin genes (**e**) from different developmental stages in *Eupeodes corollae*. **e** Each data block represents the base 10 logarithm of FPKM (log_10_ FPKM) value of the corresponding samples

Because the larvae of *E. corollae* feed mainly on aphids and adults mainly on pollen, we compared the expression levels of digestive genes between eggs and larvae, larvae and pupae, pupae and adults, and larvae and adults. Compared to the genes in the eggs, most genes were significantly upregulated in larvae after they had fed on aphids, consistent with their roles in aphid digestion (Fig. 4a). In pupae compared to larvae, most digestive-related genes were downregulated (Fig. 4b). Because the adults feed on pollen or nectar, most digestive-related genes were upregulated in adults compared to pupae (Fig. 4c), suggesting that digestion mainly occurs in the larvae and adults. Compared to larvae, almost all (9 of 10) trypsins were downregulated in adults, while most other SPs (15 of 26) and phospholipase (4 of 5) and all 10 opsins and four carboxylesterase were upregulated in adults (Fig. 4d, Additional file 2: Table S10). We further compared the expression profiles of trypsin genes at different developmental stages. The results showed that nine trypsin genes (*Ecor12299*-*Ecor12303*, *Ecor12307*, *Ecor13436*, *Ecor17954*, *Ecor18958*) were significantly upregulated in first- to third-instar larvae and downregulated in adults (Fig. 4e), suggesting these genes might be involved in digestion and absorption of aphids.

In the comparative genomic analysis between *E. corollae* and *S. pyrastri*, four protease genes (*Ecor12299*, *Ecor12301*, *Ecor2966*, *Ecor7242*) were identified as homologous genes in the two species (Fig. 2), and the expression levels of these protease genes were analyzed. The results showed that of the four trypsin genes, all but *Ecor7242* were expressed strongly in larvae (Fig. 4e) and likely to be essential for digesting aphids in *E. corollae*.

### The genomic basis of foraging behavior

As a predator of aphids and a pollinator, *E. corollae* relies on its chemoreception system to perceive chemical cues from its prey insects and flowering plants to mediate behaviors such as prey foraging, feeding, mating, oviposition, and pollination [5, 24–26]. In the genome of *E. corollae*, 36 gustatory receptors (GRs), 46 odorant receptors (ORs), 36 ionotropic receptors (IRs), four sensory neuron membrane proteins (SNMPs), four chemosensory proteins (CSPs), and 46 odorant-binding proteins (OBPs) were manually identified (Table 2). Fewer chemosensory genes were found in *E. corollae* than in other dipteran species [27, 28], which might be related to the narrow food habits of *E. corollae*.

**Fig. 5:**
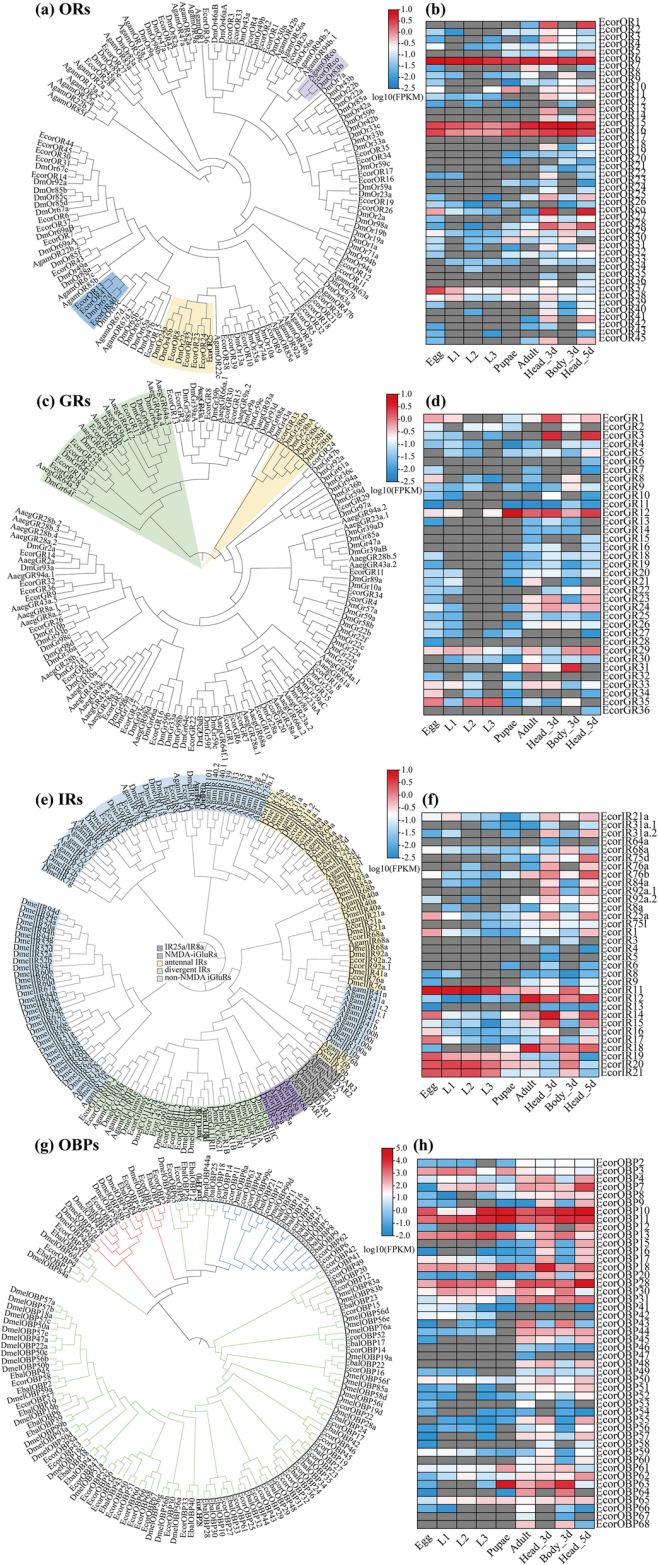
Chemosensory-related genes in *Eupeodes corollae*. Phylogenetic tree of odorant receptors (ORs) (**a**), gustatory receptors (GRs) (**c**), ionotropic receptors (IRs) (**e**), and odorant-binding proteins (OBPs) (**g**) from *E. corollae* and other dipteran species. Predicted genes in *E. corollae* are indicated by different colors. **a** ORco (purple), pheromone receptors (blue), tandem repeats (yellow). **c** GR64 subfamily (green) and GR28 subfamily (yellow). **e** IR25a/IR8a (purple), antennal IRs (yellow), divergent IRs (blue), NMDA-iGluRs (gray), and non-NMDA iGluRs (green). **g** Classic OBPs (green), Minus-C (blue), and Plus-C (red). Species for this phylogeny included *E. corollae* (Ecor), *Drosophila melanogaster* (Dm), *Anopheles gambiae* (Agam), *Aedes aegypti* (Aaeg), and *Episyrphus balteatus* (Ebal). Expression profile of ORs (**b**), GRs (**d**), IRs (**f**), and OBPs (**h**) for different developmental stages of *E. corollae*. Each data block represents the base 10 logarithm of FPKM (log_10_ FPKM) value of the corresponding samples

ORs are seven-transmembrane domain proteins, and their encoding genes are expressed in olfactory sensory neurons (OSNs) for selectively sensing volatile chemicals in the environment [29, 30]. The number of OR-encoding genes identified in the genome assembly (46) is close to that in the previously reported transcriptome of *E. corollae* (42) and *E. balteatus* (51), but fewer than in *D. melanogaster* (62), *A. gambiae* (79), and *A. aegypti* (131) (Table 2) [20, 28, 31]. Further phylogenetic analysis showed that three EcorORs (EcorOR13, 40, 41) in *E. corollae* clustered with the pheromone receptor DmelOR67d [32], while EcorOR7 clustered with DmelOR69aB, suggesting these genes might be implicated in important roles in pheromone recognition in *E. corollae* (Fig. 5a). However, the homologous genes to other pheromone receptors DmelOR88a or DmelOR65a were not found in *E. corollae*.

Spatial and temporal expression of ORs showed that ORs were mainly expressed in the adult head at 3 and 5 days after eclosion (Fig. 5b), suggesting these genes might play important roles in mating and oviposition behaviors of *E. corollae*. In addition, three OR genes (EcorOR6, 15, 16) were highly expressed throughout development (egg to adult). Previous researches mainly focused on ORs that are highly expressed in the antennae of insects [33, 34]. However, ORs also have other important biological functions in non-head tissues in insects. For example, *A. gambiae* ORs are expressed strongly in the testes and function in sperm activation [35]. Thus, we speculated that these three ORs might have basic physiological functions in *E. corollae*.

GRs are mainly expressed at gustatory receptor neurons for sensing non-volatile chemicals, including sugars, bitter compounds, and carbon dioxide (CO_2_) [19, 20]. The number of GRs in *E. corollae* (36) was twofold higher than reported by Wang et al. (16) through transcriptome sequencing (Table 2). Phylogenetic analysis showed that six GRs genes clustered with the GR64 subfamily of *D. melanogaster*, which participate in sugar recognition (Fig. 5c). The expression profile analysis showed that seven GR genes were expressed at the adult stage, while two GRs were highly expressed at the larval stage (Fig. 5d).

IRs, which belong to the ionotropic glutamate receptor superfamily (iGluRs), were first found in *D. melanogaster* [36]. IRs can be divided into two subfamilies: conserved “antennal IRs” and species-specific “divergent IRs,” which function in diverse processes, including olfaction reception, taste sensing, and temperature and moisture detection [37–39]. More IRs were found here in *E. corollae* than reported by Wang et al. but similar to the 32 reported for *E. balteatus* [25]. Phylogenetic analysis showed that candidate antennal IRs clustered with “antennal” orthologues of *D. melanogaster* [38]. Homologs of DmIR68a were identified in our genome assembly, which were not found in a previous study [25]. Thirteen IR genes that clustered with the DmeliGluRs clade were identified as iGluRs of *E. corollae* (Fig. 5e). When these results are considered with the fact that the candidate antennal IR genes were mainly expressed in adult heads, then these IRs likely have olfactory functions; the other IRs had diverse expression patterns during development (Fig. 5f).

Besides chemosensory receptors, other chemosensory proteins, including OBPs, CSPs, and SNMPs, were also encoded by genes in the *E. corollae* genome. OBPs are involved in initial olfactory recognition by binding and transporting external odor molecules to the corresponding membrane receptors [40, 41]. We identified 46 OBPs encoded in the genome assembly, 18 of which were identified by Jia et al. The other 28 OBPs were named EcorOBP41–EcorOBP68. Phylogenetic analysis revealed that OBPs of *E. corollae* clustered with high bootstrap support into three clades: 34 classic, 4 plus-C, and 7 minus-C (Fig. 5g). Transcriptomic analysis showed that many OBPs (17 of 46) were highly expressed in adult heads (Fig. 5h).

### Genomic basis of detoxification

**Fig. 6:**
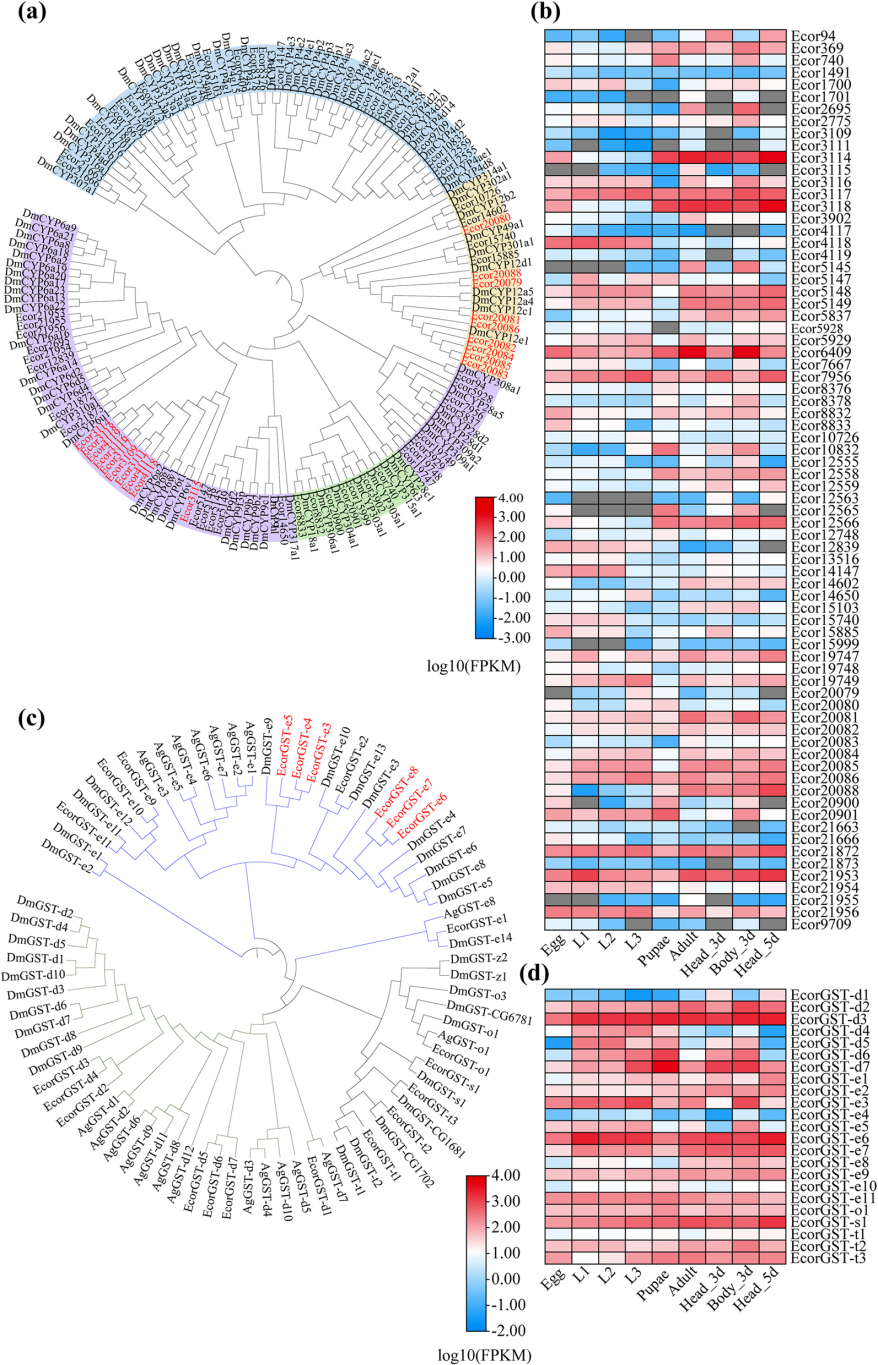
Phylogenetic tree and expression patterns of cytochrome P450s and glutathione *S*-transferases (GSTs) for different developmental stages and tissues of *Eupeodes corollae*. **a** Different colors on the clade indicate different CYP clans: CYP2 clan (green), CYP3 clan (purple), CYP4 clan (blue), and Mito clan (yellow). Tandem proteins are in red. **b** Inner branches in different colors represent different protein classes: green, delta class; blue, epsilon class. Tandem proteins are in red. Species in this phylogeny include *E. corollae* (Ecor), *Drosophila melanogaster* (Dm), and *Anopheles gambiae* (Ag). Expression patterns of cytochrome P450s (**c**) and glutathione *S*-transferases (GSTs) (**d**) for different developmental stages and tissues of *E. corollae*

Detoxification enzymes are important for metabolizing natural toxins and synthetic insecticides in insects [42, 43]. Our manual annotation of detoxification-related genes included 74 cytochrome P450s and 27 glutathione *S*-transferases (GSTs) in the *E. corollae* genome. P450s are phase I detoxification enzymes involved in the metabolism of a wide range of endogenous and exogenous compounds [44]. *E. corollae* was predicted to have fewer P450s than *D. melanogaster* (85) and other dipteran species (Table 2) [45, 46]. Phylogenetic analysis indicated 10 genes (*Ecor3109*, *Ecor3111*, *Ecor3114*–*Ecor3118*, *Ecor4117*–*Ecor4119*) from the CYP3 clade, and 9 genes (*Ecor20079*–*Ecor20086*, *Ecor20088*) from the mitochondrial P450 clade were arranged in tandem in *E. corollae* genome (Fig. 6a, Additional file 1: Fig. S4). Nine expanded genes (*Ecor3109*, *Ecor3111*, *Ecor3114*, *Ecor3116*–*Ecor3118*, *Ecor4117*–*Ecor4119*) clustered with DmCYP6G2, which can metabolize insecticides (e.g., imidacloprid) and confer insecticide resistance to *D. melanogaster* [47, 48], suggesting that these proteins might contribute to the detoxification capacity of *E. corollae*. Based on the transcriptomic analysis, the expression of P450 genes differed among developmental stages and tissues, indicating diverse functions for the P450s (Fig. 6b).

GSTs are multifunctional enzymes in phase II detoxification [49]. The 27 putative GST genes identified in *E. corollae* encoded 23 cytosolic GSTs and four microsomal GSTs. Phylogenetic analysis showed that the 23 cytosolic GSTs were classified into five classes, with seven in delta, 11 in epsilon, one in omega, one in sigma, and three in theta (Fig. 6c). The delta and epsilon classes had the most members, which were insect-specific and involved in resistance to pesticides such as organophosphates and organochlorines [50–52]. Six genes from the epsilon class (EcorGSTe3-EcorGSTe8) were arranged in tandem. All GSTs were expressed at different levels at different developmental stages and in different tissues of *E. corollae* (Fig. 6d).

## Discussion

The genome size of the assembly presented here for the hoverfly *E. corollae* was 595 Mb, close to the estimated genome size by 17-mer analysis (604 Mb), suggesting the assembly in our study was appropriate. We then compared this genome with those of insects with similar biological characteristics: aphidophagous hoverfly *S. pyrastri*, aphid predator ladybugs, and pollinator honeybees [53] to elucidate the genetic basis of predation and pollination. These comparative analyses revealed a number of genes in *E. corollae* that are strongly linked to digestion, feeding behavior, chemoreception, sugar symporter activity, and lipid transport, such as genes for trypsin, takeout, GRs, trehalose transporters, and phospholipid-transporting ATPase, which are important for predation and pollination [19–21]. Transcriptomic analysis revealed that 10 opsin genes, which are involved in visual perception [54], were significantly upregulated in adults. These findings expand our understanding of adaptations for predation and pollination in the hoverfly *E. corollae*.

*E. corollae* digests aphids as the primary food source of larvae, and the diversity of its digestive enzymes should approximately match the composition of its diet as found for other insects [55]. For example, fewer genes related to digestion were identified in the brown planthopper, *Nilaparvata lugens*, which has a simple diet, phloem sap [56]. In our study, *E. corollae* also had fewer digestion-related genes compared with other dipteran species, also likely due to its simple aphid diet, in contrast to the broad diet of polyphagous insect species [38]. For example, SPs are significantly abundant in the omnivorous pest *A. lucorum* [38]. In addition, insects can regulate the expression of digestive enzymes homeostatically. In *Drosophila*, the activity of amylase in larvae is significantly higher when they feed on starch diets compared with sugar diets [57]. Our transcriptomic sequencing showed that more trypsins were highly expressed in larvae of *E. corollae*, consistent with the fact that aphid composition is more complex, including proteins, starches, and lipids, compared to the adult diet of sugar-rich nectar. Comparative genomic analyses of *E. corollae* and other aphidophagous hoverflies highlighted three homologous trypsins and their strong expression at the larval stage additionally supported their potential role in aphid digestion. In addition, microbial endosymbionts, mainly bacteria, might also have important roles in nutrient metabolism [58, 59], which will be examined in further research.

In summary, we have provided insights into the genetic basis of predation and pollination by *E. corollae*, an efficient aphid predator. The chromosome-level genomic and transcriptomic data for *E. corollae* are valuable resources for advancing studies on genetic adaptations, evolution, and its use as a beneficial insect.

## Conclusions

*E. corollae* and other hoverflies (Diptera: Syrphidae) are important pollinators of many plants and promising biological control agents for controlling aphid pests worldwide. In this study, we present a chromosome-level genome assembly of the hoverfly *E. corollae* to elucidate the genetic basis of predatory adaptation and pollination in insects. Comparative genomic analysis shed light on three *takeout* genes, which are related to circadian rhythms and feeding behavior and induced by starvation. Genes for sugar symporter and lipid transport involved in sugar transport and energy production were also present in *E. corollae* similar to the genome of honeybees, reflecting the important pollinator role of hoverflies. Seven P450s from the cytochrome CYP6 subfamily were expanded in the *E. corollae*, which might improve detoxification capacity. Furthermore, comparative genomic analysis between *E. corollae* and *S. pyrastri* identified four trypsins, three of which (Ecor12299, Ecor12301, Ecor2966) were expressed strongly in larvae, supporting their role in aphid digestion by *E. corollae*. These results of *E. corollae* lay the foundation for in-depth research of *E. corollae* and analyses of predation and pollination in hoverflies and other natural enemies.

## Materials and methods

### Sample preparation and genome sequencing

*E. corollae* adults were collected in Langfang, Hebei Province, China, in 2015 and reared in the lab at 23 ± 1 °C with 14 h light:10 h dark. After egg hatching, the larvae were fed with aphids on bean plants, and emerging adults were provided with pollen and honey [22]. An inbred strain (Ec2018), produced by single-pair sib matings for five generations, was used to sequence the genome and transcriptome. For PacBio sequencing, genomic DNA was extracted from a pooled sample of five female adults. A long library with an insert size of ~ 20 kb was constructed and sequenced on six cells using a PacBio RS II system (Pacific Biosciences). A DNA library with a short insert size (400–500 bp) from one female adult was constructed without PCR and sequenced using an Illumina HiSeq X Ten platform. We obtained a 17 k-mer depth distribution using Jellyfish [60] based on the Illumina data and estimated the size and heterozygosity of the *E. corollae* genome using GenomeScope [61].

The Hi-C library was constructed using 10 female adults. The sample was fixed with 2% v/v formaldehyde for cross-linking. After cross-linking completely, the sample was lysed. The chromatin was digested with the restriction enzyme DpnII and labeled with biotin and ligated. DNA was extracted and purified to obtain a Hi-C sample. After biotin-removed, blunt end-repaired, A-tailed, and adaptor ligation, the Hi-C library was amplified by PCR to obtain the library products. Hi-C libraries were constructed and sequenced on an Illumina NovaSeq platform.

### Transcriptomic sequencing and analysis

Samples at different developmental stages (30 eggs, 30 first instar larvae, 3 s instar larvae, 3 third instar larvae, 3 pupae, and 3 adults per group) and tissues from female adults (including 3-day-old heads, 3-day-old bodies, and 5-day-old heads, *n* = 3 per group) were collected and used to extract total RNA using TRIzol Reagent (Invitrogen). The purity and concentration were determined with a NanoDrop 2000 spectrophotometer (Thermo Scientific) and 4200 Bioanalyzer (Agilent), respectively. Then, cDNA libraries were constructed using high-quality RNA and sequenced using an Illumina NovaSeq platform. There were three groups for each sample.

After sequencing, raw reads were first filtered by removing adaptor, duplicated, and low-quality sequences. The resulting clean reads were aligned with the *E. corollae* genome assembly using HISAT2 [62]. The transcript levels of genes in each sample were quantified using HiSeq and normalized to fragments per kilobase per million reads (FPKM) values. Then, edgeR [63] was used for differential expression analysis of genes. Genes with a false discovery rate (FDR) < 0.05 and log_2_ |FoldChange|> 1 were considered as differentially expressed [64].

### Genome assembly

The adapter and low-quality sequences of Illumina raw reads were trimmed using in-house software clean_adapter (version 1.1) and clean_lowqual (version 1.0) to generate clean reads. The PacBio raw reads were initially processed to correct errors and trim short reads (< 5 kb) using CANU (version 1.8) [65]. Then, PacBio clean reads were used for contig genome assembly with wtdbg2 (version 2.4) [15]. To polish the genome, we aligned the PacBio raw reads with the assembly and corrected errors using FinisherSC (version 2.1) [66] (https://github.com/kakitone/finishingTool). In addition, the Illumina clean reads were aligned with the genome using bowtie2 (version 2.4.1) [67] (https://github.com/BenLangmead/bowtie2), and single-base errors were corrected using pilon (version 1.23) [68] (https://github.com/broadinstitute/pilon). We mapped the Illumina clean reads to the genome assembly to calculate the mapping rate and the depth of genome coverage using BWA (version 0.7.12) [69]. The completeness of the genome was assessed using BUSCO (version 3.1.0) by searching against insecta_odb9 data sets.

After quality control, the Hi-C library was constructed and sequenced using the Illumina NovaSeq system and PE150 strategy. After sequencing, adapter and low-quality sequences were filtered out from Hi-C raw reads. The resulting high-quality reads were then mapped to the genome with BWA (version 0.7.12), and invalid read pairs were filtered. The valid Hi-C data were used for scaffolding the contig assembly using ALLHiC [70] with default parameters (except for -e GATC -k 4).

### Gene prediction and annotation

Repeat sequences in the assembly were predicted using two methods: homology-based and de novo predictions. RepeatMasker (version 4.0.3) was used for homology-based predictions with the Repbase library. A de novo repeat database for *E. corollae* was built for de novo predictions using RepeatModeler (version 1.0.8).

Based on the repeat-masked genome, we predicted gene models by combining evidences from de novo gene prediction, homology searching, and transcriptome sequencing using BRAKER2 (version 2.1.5). For RNA-seq annotation, six data sets from the different developmental stages were mapped to the genome using STAR v2.7.1a with default parameters [71]. For homology searches, proteins from the NCBI Diptera UniRef50 database were aligned to the *E. corollae* genome by GenomeThreader v1.7.1 [72]. Based on the alignment results, GeneMark-ET [73] was used to generate the initial gene structures. Then, AUGUSTUS v2.5.5 [74] was used to produce the final gene predictions using the initial gene models. The protein sequences of predicted genes were used in searches of the Swiss-Prot, NR, eggNOG, and KEGG databases for functional annotation using DIAMOND (version 0.8.28) with an *e*-value cutoff of 1e − 5.

### Comparative genomics analysis

Protein sequences of 15 representative dipteran species with high-quality genomes including *A. aegypti*, *Anopheles darlingi*, *A. gambiae*, *Anopheles sinensis*, *Bactrocera dorsalis*, *Ceratitis capitata*, *Culex quinquefasciatus*, *D. melanogaster*, *Lucilia cuprina*, *Musca domestica*, *Stomoxys calcitrans*, *Eristalis dimidiate*, *Eristalis tenax*, *S. pyrastri*, and *Syritta pipiens*, and three coleopteran species including *C. septempunctata*, *H. axyridis*, and *P. japonica*. Hymenopteran species *A. mellifera* and *A. cerana* were used as outgroups. All sequences for the comparative analyses were downloaded from NCBI databases. Redundant alternative splicing events were filtered to keep the longest transcript for each gene. OrthoFinder v2.3.1 [75] was adopted to identify orthologous and paralogous genes. Protein sequences of single-copy genes were used for multiple sequence alignments using MAFFT v7 [76]. TrimAL v1.2 [77] was used to trim sequences, extract the conserved region, and concatenate all single-copy genes into a super-sequence, which was used for a maximum likelihood (ML) tree construction. The phylogenetic analysis was performed using IQ-TREE (version 1.5.5) with model selection across each partition and 1000 ultrafast bootstrap replicates. The divergence time was estimated using r8s (version 1.81) [78] based on fossil calibration points. The estimated divergence time between *A. aegypti* and *C. quinquefasciatus* was 75 Mya and 37 Mya between *M. domestica* and *S. calcitrans*.

Orthologous groups of each species were generated by OrthoFinder with default parameters. We manually identified the predicted orthogroups between *E. corollae* and *S. pyrastri*, which were not found in other species. To predict genes related to predation, the manually curated orthogroups between *E. corollae* plus three ladybugs did not contain honeybee homologs. Similarly, to predict genes related to pollination, we manually identified homologous genes shared by *E. corollae* plus two honeybees, which were absent from ladybugs. The homologous genes were further used for GO enrichment analysis for functional annotation.

### Gene family analysis

We manually annotated detoxification-related and chemosensory-related gene families. For these gene families, protein sequences of dipteran species were downloaded from NCBI and aligned with the *E. corollae* genome using TBLASTN (*e*-value = 1e − 5). Then, hidden Markov models (HMMs) of P450s (PF00067), GST (PF13417, PF02798, PF00043, PF14497, or PF13410), IRs (PF10613 or PF00060), GRs (PF06151 or PF08395), ORs (PF02949 or PF13853), OBPs (PF01395), CSPs (PF03392), and SNMPs (PF01130) were downloaded from the Pfam database, and HMMER (version 3.3) was used to identify the candidate genes [79]. A neighbor-joining (NJ) phylogenetic tree for each gene family was constructed in MEGA7 [80] with 1000 bootstrap replicates.

## Supplementary Information

**Additional file 1:** **Table S1.** Statistics for sequencing data. **Table S2.** Summary of statistics for *Eupeodes corollae* chromosomes. **Table S3.** BUSCO (Benchmarking Universal Single-Copy Orthologues) assessment of *Eupeodes corollae* genome using insecta_odb9 data sets (*n* = 1,658). **Table S4.** Characteristics of transposable elements in *Eupeodes corollae*. **Fig. S1. **Distribution of 17-mer frequency of Illumina sequencing reads of *Eupeodes corollae*. **Fig. S2. **Venn plot of functional annotations for predicted proteins of *Eupeodes corollae*. **Fig. S3. **The number of the orthologous groups shared between *Eupeodes corollae* and other species by OrthoFinder analysis. **Fig. S4. **Distribution of cytochrome P450 genes on the four chromosomes of *Eupeodes corollae*.

**Additional file 2:** **Table S5. **GO enrichment analysis of Syrphidae-specific genes (Fisher’s exact test, *p* < 0.05). **Table S6. **GO enrichment analysis of species-specific genes of *Eupeodes corollae* (Fisher’s exact test, *p* < 0.05). **Table S7.** GO enrichment analysis of the homologous genes shared between *Eupeodes corollae* and *Scaeva pyrastri* (Fisher’s exact test, *p* < 0.05). **Table S8. **GO enrichment analysis of the homologous genes shared between *Eupeodes corollae* and ladybugs (Fisher’s exact test, *p* < 0.05). **Table S9.** GO enrichment analysis of the homologous genes shared between *Eupeodes corollae* and honeybees (Fisher’s exact test, p < 0.05). **Table S10.** Differentially expressed genes in adults compared to larvae stage in *Eupeodes corollae*.

## Data Availability

Data supporting the findings of this work are available within the paper and supplementary information files. All the raw sequencing data and genome data in this study have been deposited at NCBI as a BioProject under accession PRJNA746055 [81]. Genomic sequence reads have been deposited in the SRA database as BioSample SAMN20179301 [82]. Transcriptome sequence reads have been deposited in the SRA database as BioSample SAMN20169051 [83]. This Whole Genome Shotgun project has been deposited at DDBJ/ENA/GenBank under accession JAIWPZ000000000 [84]. The version described in this paper is version JAIWPZ010000000.

## Abbreviations

BUSCO: Benchmarking Universal Single-Copy Orthologs
LINEs: Long interspersed elements
SPs: Serine proteases
OBPs: Odorant-binding proteins
CSPs: Chemosensory proteins
ORs: Odorant receptors
IRs: Ionotropic receptors
GRs: Gustatory receptors
SNMPs: Sensory neuron membrane proteins
GSTs: Glutathione *S*-transferases
OSNs: Olfactory sensory neurons
iGluRs: Ionotropic glutamate receptor superfamily
FPKM: Fragments per kilobase per million reads
FDR: False discovery rate
GO: Gene Ontology
HMMs: Hidden Markov models
