# Supplementary material for: Genome of the hoverfly Eupeodes corollae provides insights into the evolution of predation and pollination in insects
Source: BMC Biol. 2022 Jul 6;20:157. doi: 10.1186/s12915-022-01356-6 (PMC9261035; doi:10.1186/s12915-022-01356-6)
Supplement: Supplementary file 1 — Additional file 1: Table S1. Statistics for sequencing data. Table S2. Summary of statistics for Eupeodes corollae chromosomes. Table S3. BUSCO (Benchmarking Universal Single-Copy Orthologues) assessment of Eupeodes corollae genome using insecta_odb9 data sets (n = 1,658). Table S4. Characteristics of transposable elements in Eupeodes corollae. Fig. S1. Distribution of 17-mer frequency of Illumina sequencing reads of Eupeodes corollae. Fig. S2. Venn plot of functional annotations for predicted proteins of Eupeodes corollae. Fig. S3. The number of the orthologous groups shared between Eupeodes corollae and other species by OrthoFinder analysis. Fig. S4. Distribution of cytochrome P450 genes on the four chromosomes of Eupeodes corollae. [file 12915_2022_1356_MOESM1_ESM.docx]

**Genome of the hoverfly *Eupeodes corollae* provides insights into evolution of predation and pollination in insects**

He Yuan^1, 2^, Bojia Gao^2^, Chao Wu^2^, Lei Zhang^2^, Hui Li^3^, Yutao Xiao^2^, Kongming Wu^1^

Table S1. Statistics for sequencing data.

| Source |  | Total bases | No. reads | Clean bases | Clean reads |
| --- | --- | --- | --- | --- | --- |
| Pacbio data (6 cells) | 1 | 12,208,926,838 | 711,016 | 12,191,850,495 | 1,092,747 |
|  | 2 | 12,979,210,478 | 787,540 | 12,963,477,628 | 1,126,624 |
|  | 3 | 11,659,271,164 | 755,697 | 11,645,336,699 | 1,060,153 |
|  | 4 | 12,083,534,678 | 775,555 | 12,068,828,357 | 1,091,366 |
|  | 5 | 10,140,353,595 | 638,043 | 10,130,579,376 | 854,801 |
|  | 6 | 6,777,141,970 | 530,733 | 6,770,848,195 | 666,776 |
|  | Total | 65,848,438,723 | 4,198,584 | 65,770,920,750 | 5,892,467 |
| Illumina DNA data |  | 60,356,278,200 | 201,187,594 | 60,228,662,100 | 200,762,207 |
| Illumina RNA-Seq data | Egg |  |  | 20,450,089,200 | 68,166,964 |
|  | L1 |  |  | 21,315,127,200 | 71,050,424 |
|  | L2 |  |  | 20,302,810,800 | 67,676,036 |
|  | L3 |  |  | 22,537,971,300 | 75,126,571 |
|  | Pupae |  |  | 25,062,220,500 | 83,540,735 |
|  | Adult |  |  | 20,670,013,800 | 68,900,046 |
|  | Head_3d |  |  | 20,481,093,600 | 68,270,312 |
|  | Body_3d |  |  | 21,293,537,700 | 70,978,459 |
|  | Head_5d |  |  | 23,416,042,500 | 78,053,475 |
|  | Total |  |  | 195,528,906,600 | 651,763,022 |
| Illumina Hi-C |  |  |  | 55,424,590,448 | 369,964,038 |

Table S2. Summary of statistics for *Eupeodes corollae* chromosomes.

| Chromosome | Contig length (bp) | Contig number |
| --- | --- | --- |
| Chr1 | 334,938,377 | 311 |
| Chr2 | 106,405,447 | 200 |
| Chr3 | 104,039,156 | 651 |
| Chr4 | 25,456,275 | 187 |
| Total anchored | 570,839,255 | 1349 |
| Unanchored | 24,711,222 | 1897 |

Table S3. BUSCO (Benchmarking Universal Single-Copy Orthologues) assessment of *E. corollae* genome using insecta_odb9 datasets (*n* = 1,658).

|  | Assembly | | Annotation | |
| --- | --- | --- | --- | --- |
| Term | Number | Rate | Number | Rate |
| Complete BUSCOs | 1610 | 97.1% | 1608 | 97.0% |
| Complete and single-copy BUSCOs | 1530 | 92.3% | 1454 | 87.7% |
| Complete and duplicated BUSCOs | 80 | 4.8% | 154 | 9.3% |
| Fragmented BUSCOs | 20 | 1.2% | 22 | 1.3% |
| Missing BUSCOs | 28 | 1.7% | 28 | 1.7% |
| Total BUSCO groups searched | 1658 |  | 1658 |  |

Table S4. Characteristics of transposable elements in *E. corollae*. LINEs, long interspersed nuclear elements; SINEs, short interspersed nuclear elements. LTR, long terminal repeats.

| Elements | No. of elements* | Length (bp) | Percentage of sequence (%) |
| --- | --- | --- | --- |
| SINEs | 0 | 0 | 0.00 |
| LINEs | 253,384 | 139,061,082 | 23.35 |
| LTR elements | 10,861 | 7,077,567 | 1.19 |
| DNA elements | 219,463 | 64,877,003 | 10.89 |
| Unclassified | 326,855 | 87,980,030 | 14.77 |
| Simple repeats | 153,137 | 7,455,541 | 1.25 |
| Satellites | 0 | 0 | 0.00 |
| Small RNA | 320 | 34,473 | 0.01 |

**Supplemental figures**


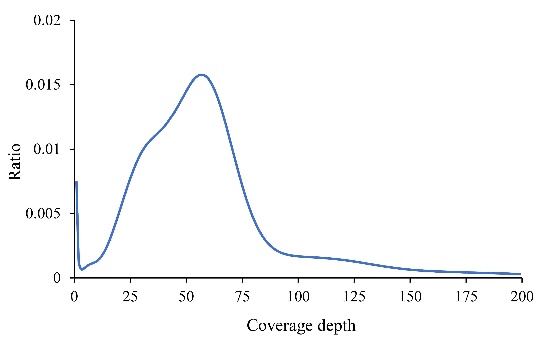


**Fig. S1. Distribution of 17-mer frequency of Illumina sequencing reads of *Eupeodes corollae*.** The peak depth of this curve was 58. The estimated genome size was 604 Mb. The heterozygosity ratio was estimated to be 0.84%.


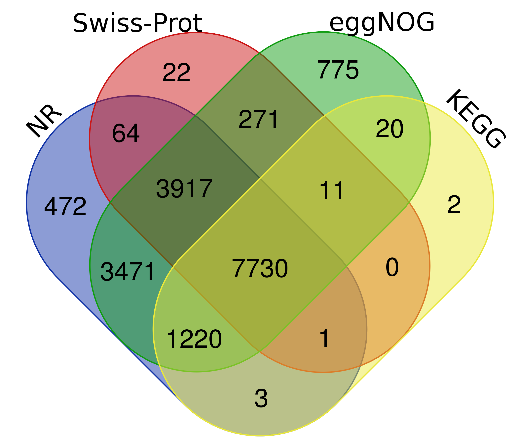


**Fig. S2. Venn plot of functional annotations for predicted proteins of *Eupeodes corollae*.** Figure was plotted using the Venn tool at <http://bioinformatics.psb.ugent.be/webtools/Venn/>. A total of 16,878 (72.21%), 12,016 (51.41%), 17,415 (74.51%), 9,042 (38.68%) genes were used in functional annotations of proteins using the NR, Swiss-Prot, eggNOG, and KEGG databases respectively. In total, 17.979 (76.92%) predicted proteins have homology in either of these databases.


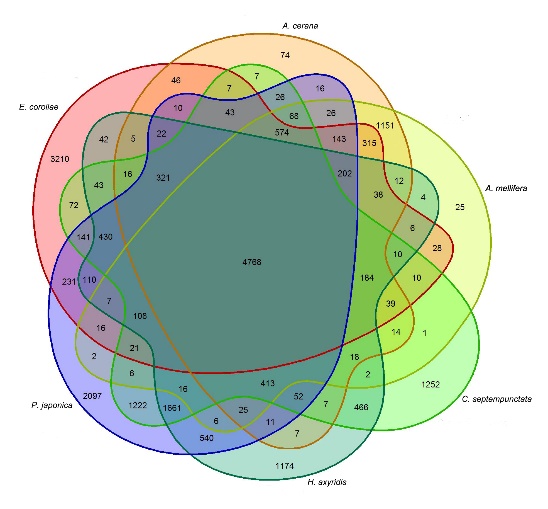


**Fig. S3. The number of the orthologous groups shared between *Eupeodes corollae* and other species by OrthoFinder analysis.**

**
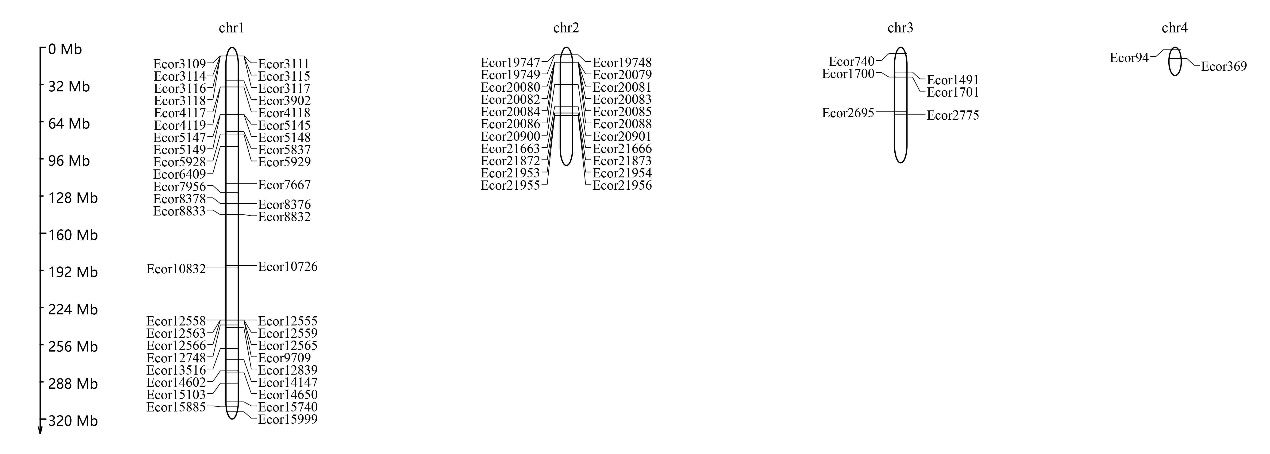
**

**Fig. S4. Distribution of cytochrome P450 genes on the 4 chromosomes of *Eupeodes corollae*.**
